# Evidence for paternal DNA transmission to gynogenetic grass carp

**DOI:** 10.1186/s12863-018-0712-x

**Published:** 2019-01-07

**Authors:** Zhuangwen Mao, Yeqing Fu, Yude Wang, Shi Wang, Minghe Zhang, Xin Gao, Kaikun Luo, Qinbo Qin, Chun Zhang, Min Tao, Zhanzhou Yao, Shaojun Liu

**Affiliations:** 1 State Key Laboratory of Developmental Biology of Freshwater Fish, Hunan Normal University, Changsha, 410081 Hunan People’s Republic of China; 2 College of Life Sciences, Hunan Normal University, Changsha, 410081 Hunan People’s Republic of China

**Keywords:** *Ctenopharyngodon idellus*, *Cyprinus carpio haematopterus*, Gynogenesis, Microsatellite DNA, DNA fragment

## Abstract

**Background:**

Grass carp (*Ctenopharyngodon idellus*, GC), as the highest-output fish in China, is economically important. The production of gynogenetic grass carp (GGC) will provide important germplasm resource for producing improved GC. At present, knowledge regarding the heterologous sperm DNA in gynogenetic offspring is little. Thus, revealing paternal DNA in GGC at the molecular level would be highly significant for fish genetic breeding.

**Result:**

In this study, ultraviolet-treated sperm of koi carp (*Cyprinus carpio haematopterus*, KOC, 2n = 100), was used to activate the eggs of GC (2n = 48). Afterwards, cold shock (0–4 °C) was administered for 12 min to double the chromosomes, resulting in GGC. No significant difference (*p >* 0.05) was found between GGC and GC in appearance, erythrocytes size and chromosome numbers. However, at the molecular level, a specific microsatellite DNA fragment (MFW1-gynogenetic grass carp, MFW1-G) derived from the paternal parent KOC was found to be transmitted into GGC.

**Conclusions:**

For the first time, this study provided an evidence at the molecular level that the DNA fragment derived from the paternal parent occurred in GGC. This finding is of great significance for fish genetic breeding.

## Background

Gynogenesis is a type of special reproductive strategy. Artificial gynogenesis usually uses ultraviolet (UV)-treated heterologous sperm to activate the eggs, then the eggs are treated by cold shock or heat shock to double the chromosomes, resulting in gynogenetic progenies.

Yi et al. [1] found microchromosomes from the second-generation gynogenetic gibel carp (*Carassius auratus gibelio*), in which the gynogenetic gibel carp eggs were activated by UV-treated sperm of blunt-nose black bream (*Megaloabrama amblycephala* Yin), providing the evidence at the cellular level that the genetic material from heterogenous sperm had the effects on gynogenetic offspring. But this study did not reveal the DNA sequences of the microchromosomes. Peek AS et al. [2] found the paternal genetic material in the gynogenetic albino rainbow trout (*Oncorhynchus mykiss*) activated by the UV-treated sperm of the wild type pigmented brook trout (*Salvelinus fontinalis*). However, the knowledge regarding the effects of the heterogenous sperm in the gynogenesis is little.

Taxonomically, grass carp (*Ctenopharyngodon idellus*, GC) belongs to the *Cyprinidae, Leuciscinae*, while koi carp (*Cyprinus carpio haematopterus*, KOC) belongs to the *Cyprinidae, Cyprininae*. GC not only grow quickly but also mainly eat the plant feed [3]. However, the germplasm degradation in GC has led to high disease incidence over the years. In the present study, the newly improved gynogenetic grass carp (GGC) have been formed by cold treatment to double the chromosomes of the GC eggs activated by UV-treated KOC sperm.

Microsatellites DNA, which are also called simple tandem repeats or simple sequence repeats (SSR), consist of a basic sequence containing 10–60 tandem repeats of a repeat unit of 2–6 nucleotides and two conserved flanking regions at the sides of the basic sequence, these features are distributed throughout the genome [4–7].

In this study, a pair of primers designated MFW1 (designed by Crooijmans et al. [8]) was used to amplify a specific microsatellite DNA fragment (MFW1-gynogenetic grass carp, MFW1-G), which has been identified in GGC, suggesting that the genetic material derived from the paternal parent might make the effects on the traits of gynogenetic offspring.

## Methods

### Samples

The KOC and GGC were obtained from State Key Laboratory of Developmental Biology of Freshwater Fish, Hunan Normal University, Changsha, Hunan, China; GC was obtained from Wuhu Aquatic Products, Ningxiang, Hunan, China. All the samples used in this study were cultured in ponds and artificially fed. Fish treatments were carried out according to the regulations for protected wildlife and the Administration of Affairs Concerning Animal Experimentation, and approved by the Science and Technology Bureau of China. Approval from the Department of Wildlife Administration was not required for the experiments conducted in this paper. The fish were deeply anesthetized with 100 mg/L MS-222 (Sigma-Aldrich, St Louis, MO, USA) before dissection.

### Gynogenesis

The female GC reached sexual maturity at 4 years, while the male KOC reached sexual maturity at 2 years. During the reproductive season (from May to June) in 2017 and 2018, 10 mature female GC and 10 mature male KOC were chosen as the maternal and paternal parents, respectively. The gynogenesis experiments were performed according to the methods of Liu et al. [9] and Zhang et al. [10]. First of all, the semen was diluted six times with Hank’s balanced salt solution. Then, the diluted semen was spread in culture dishes and placed under UV lamps ZW20S (Cnlight, Guangdong, China) to be sterilized (all processes were conducted in the dark). Next, the UV-treated semen of KOC was used to activate the eggs of mature female GC to develop following the cold shock (0–4 °C) for 12 min to double the chromosomes of haploid eggs. Last, the diploid eggs were incubated in water at 25 °C.

In 2017 and 2018, about 73,000 embryos were selected at random to determine the fertilization rate (number of embryos at the gastrula stage/number of eggs × 100%) and the hatching rate (number of hatched fry/number of eggs × 100%). The hatched fry were transferred to a pond for further culture.

### Measurement of morphological traits

The traits of 20 GGC, 20 KOC and 20 GC were counted and measured. The countable traits included the numbers of lateral scales, upper lateral scales, lower lateral scales, dorsal fins, abdominal fins and anal fins. The measurable traits included whole length (WL), body length (BL), body width (BW), head length (HL), head width (HW), caudal peduncle length (CPL) and caudal peduncle width (CPW). In addition, BL/WL, BW/BL, HL/BL, HW/HL, CPW/CPL and HW/BW ratios were calculated. The software of SPSS was used to analyze the covariance in the morphological traits in KOC, GC and GGC.

### Preparation of chromosome spreads

To determine the ploidy of GGC, KOC and GC, chromosome counting were performed using kidney tissues from 20 GGC, 20 KOC and 20 GC at age of 6 months. The chromosomes were prepared in accordance to the method described by Wang et al. [11]. After culturing for two to 3 days at 20–22 °C, the samples were injected one to three times with concanavalin A at a dose of 6–15 μg/g body weight at an interval of 12–24 h. Two to 3 h prior to dissection, each sample was injected with colchicine at a dose of 4–6 μg/g body weight. The kidney tissue was ground in 0.9% NaCl, subjected to hypotonic treatment with KCl (0.075 mol/L) at 37 °C for 40–60 min, and then fixed three times in 3:1 methanol-acetic acid. The cells were added dropwise onto cold, wet slides and stained with 4% Giemsa for 40–60 min. The shape and number of chromosomes were analyzed under a microscope. For each type of fish, 400 metaphase chromosome spreads (20 metaphase spreads from each sample) were analyzed. The preparations were examined under the oil lens at a magnification of 3330 × .

### Genomic DNA extraction, polymerase chain reaction (PCR) amplification, cloning and sequencing

Total genomic DNA was isolated from the tail fin in 20 GGC, 20 KOC and 20 GC using a DNA extraction kit following the manufacturer’s instructions (Sangon, Shanghai, China). A pair of primers (MFW1 F: 5’-GTCCAGACTGTCATCAGGAG-3’ and R: 5’-GAGGTGTACACTGAGTCACGC-3′) were synthesized to amplify the sequence from the mixed genomic DNA extracted from each type of fish. The PCR reactions were carried out in a volume of 30 μL with 1 μL genomic DNA (approximately 20 ng), 15 μL 2 × Taq PCR MasterMix (Tiangen, Beijing, China), 13 μL ddH_2_O and 0.5 μL of each primer. The temperature profile during amplification was initial denaturation at 94 °C for 5 min, followed by 35 cycles of 94 °C for 30 s, 53 °C for 30 s, and 72 °C for 1 min. A final extension step was performed at 72 °C for 7 min. The PCR products were separated by polyacrylamide gel electrophoresis (PAGE). The DNA fragments were purified using a gel extraction kit (UNIQ-10 Spin Column DNA Gel Extraction Kit for PAGE, Sangon) and ligated into the pMD18-T vector. The plasmids were transformed into *E.coli DH5α* and purified. The inserted DNA fragments in the pMD18-T vector were sequenced using an automated DNA sequencer (ABI PRISM 3730, Applied Biosystems, Carlsbad, CA). To analyze sequence homology and variation among the fragments amplified from GGC, KOC and GC, the sequences were aligned with BioEdit [12] and Clustal W [13]. The DNA sequences of common carp (GenBank Accession No. LN590690.1) and GC (GenBank Accession No. AY703051.1) were downloaded from the following websites:

1. The DNA sequences of common carp
https://www.ncbi.nlm.nih.gov/nuccore/LN590690.1?report=fasta
2. The DNA sequences of GC
https://www.ncbi.nlm.nih.gov/nuccore/AY703051.1

### Appearance of erythrocytes and measurement of nuclear volume

The erythrocytes of GGC, KOC, GC and red crucian carp (*Carassius auratus* red variety, RCC) were dehydrated in alcohol, added dropwise onto slides, desiccated, subjected to atomized gilding and analysed with a JSM-6360LV scanning electron microscope (SEM, JEOL, Japan). The blood smears from each fish were prepared in accordance to the method described by Lu et al. [14]. The erythrocytes from each fish were observed under oil immersion by using an ocular micrometer and the erythrocytes nuclear volume from each fish was calculated by (4/3)*πab*^2^, where *a* is the major semi-axis and *b* is the minor semi-axis of a perfect ellipsoid. The software of SPSS was used to analyze the covariance of the erythrocytes nuclear in KOC, GC and GGC.

## Results

### The basic biological characteristics

**Fig. 1:**
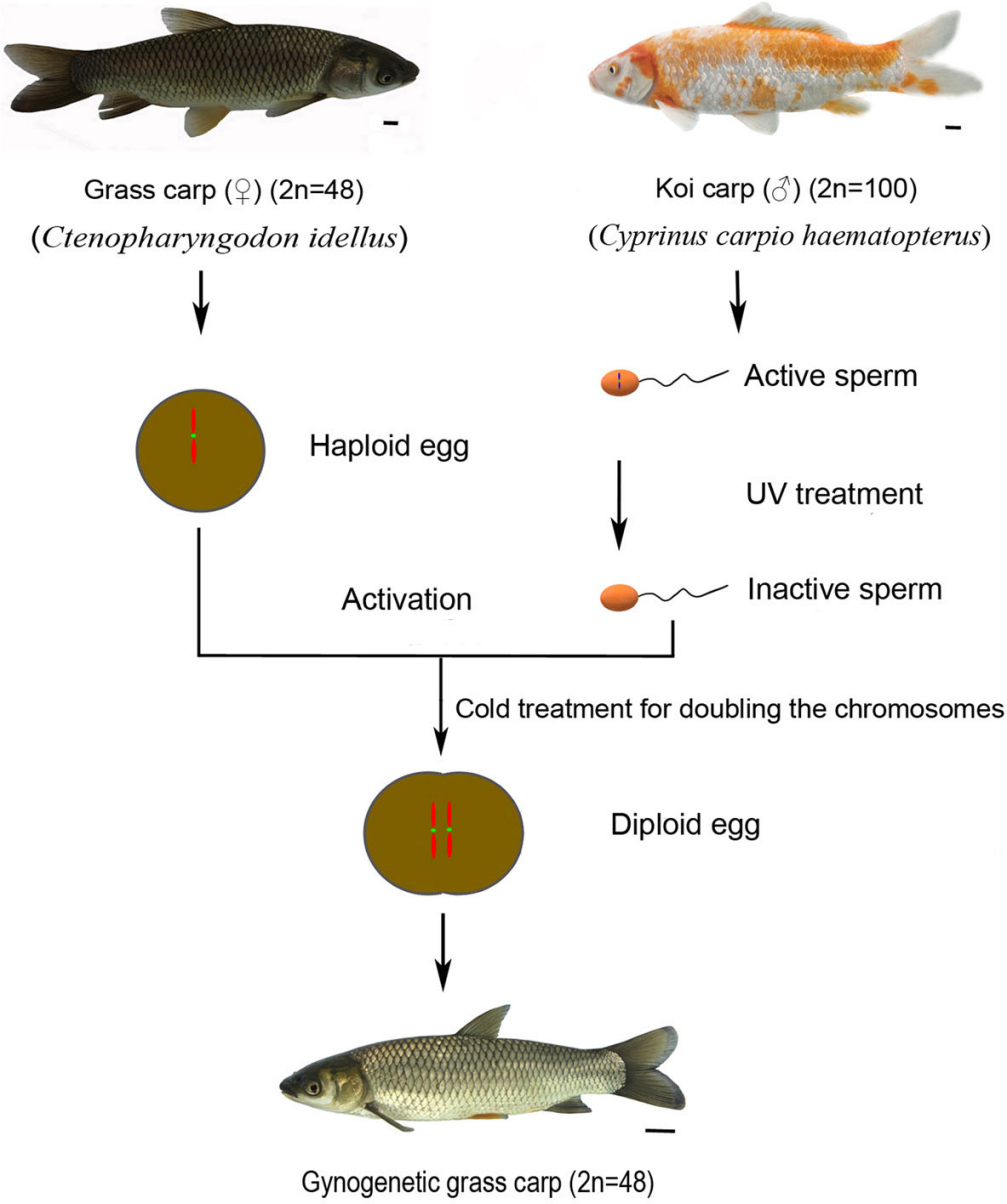
Production processes of GGC. Scale bar, 1 cm

**Fig. 2:**
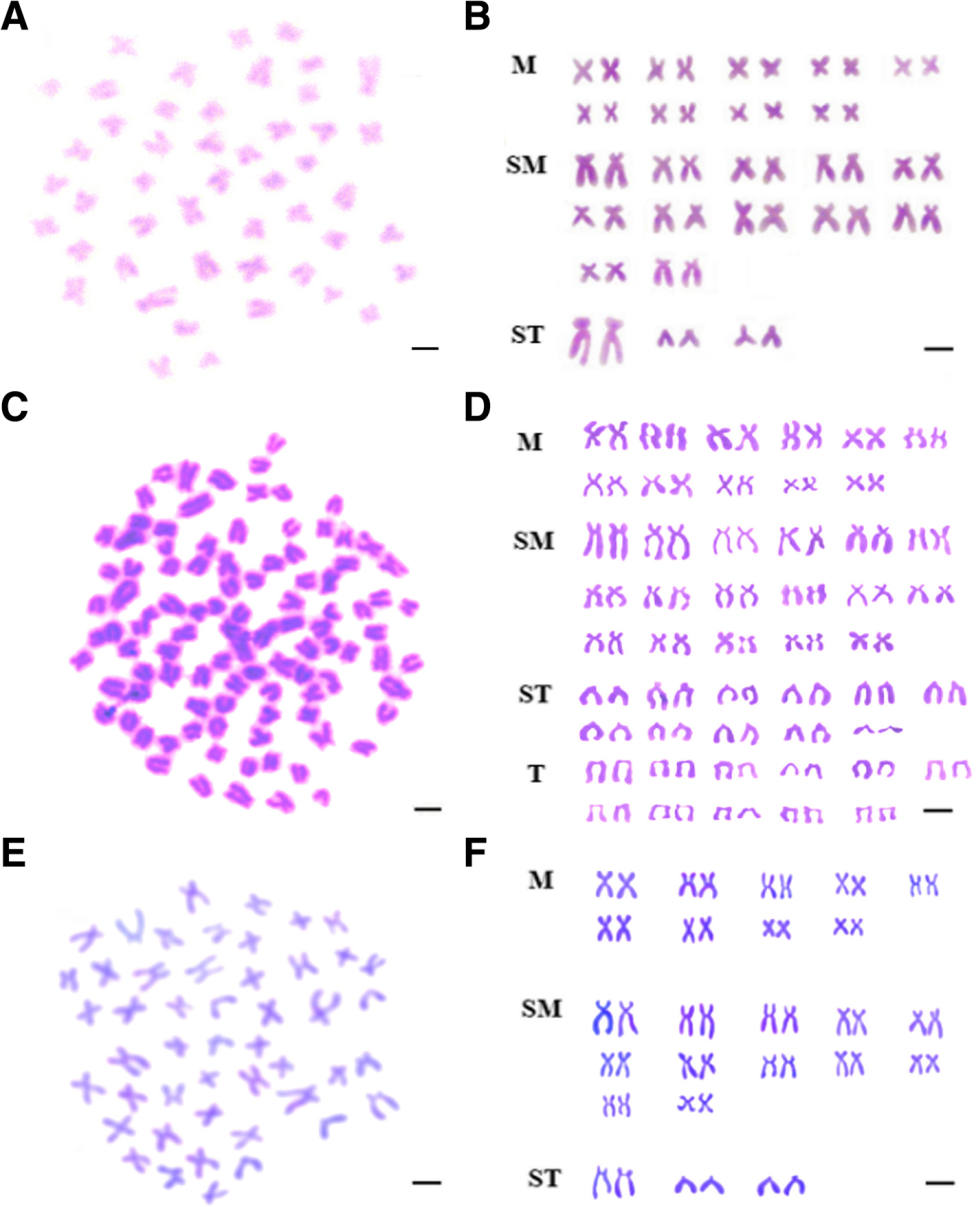
Chromosome spreads at metaphase and corresponding karyotypes of GGC, KOC and GC. **a** The 48 chromosomes of GGC; **b** The karyotype of GGC is 18 m + 24sm + 6st; **c** The 100 chromosomes of KOC; **d** The karyotype of KOC is 22 m + 34sm + 22st + 22 t; **e** The 48 chromosomes of GC; **f** The karyotype of GC is 18 m + 24sm + 6st. Scale bar, 3 μm

**Fig. 3:**
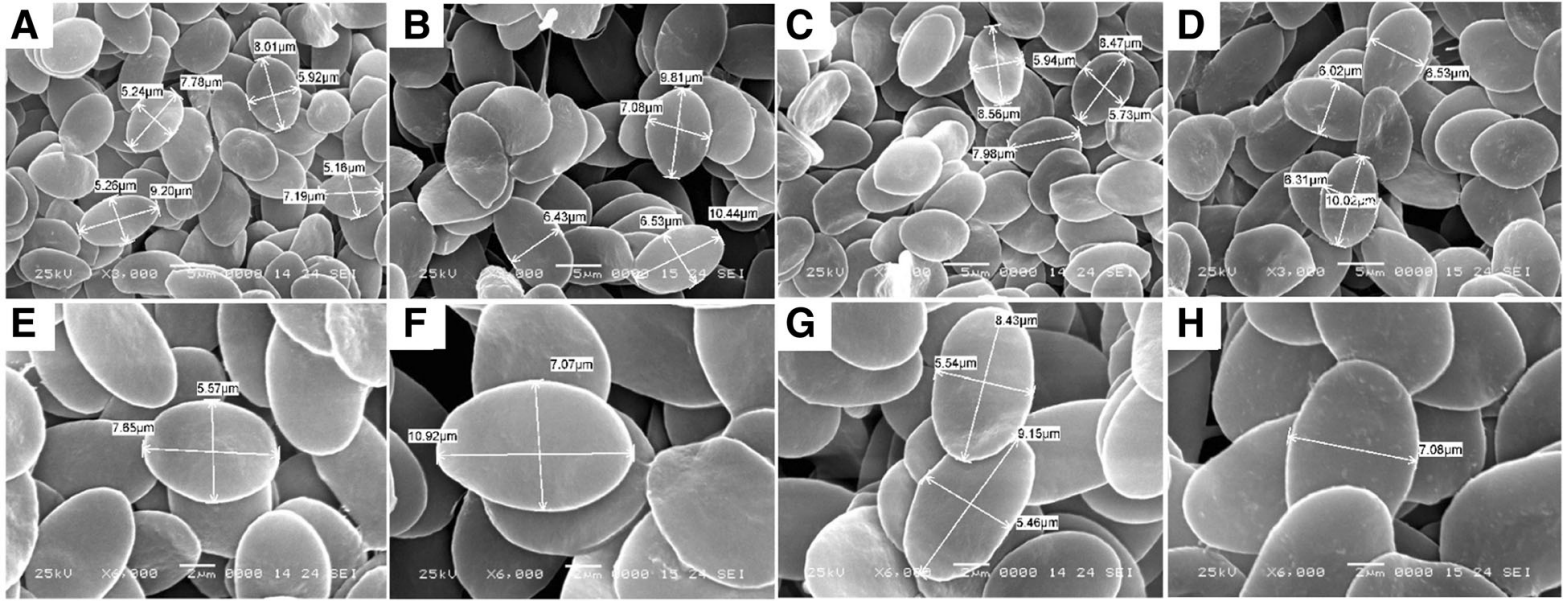
Erythrocytes of GGC, KOC, GC, and RCC under the SEM. **a**-**d** The erythrocytes of GGC, KOC, GC and RCC magnified under 3000 times, respectively. Scale bar in **a**-**d**, 5 μm; **e**-**h** The erythrocytes of GGC, KOC, GC and RCC magnified under 6000 times, respectively. Scale bar in **e**-**h**, 2 μm

The UV-treated sperm of KOC was used to activate the eggs of GC. Subsequently, the eggs were treated with cold shock (4 °C) for doubling the chromosomes, which finally developed into GGC (Fig. 1). Forty-eight chromosomes were detected in GGC with a karyotype formula of 18 m + 24sm + 6st, which was the same as that of GC (Fig. 2). Countable and measurable traits were compared among GGC, KOC and GC (Tables 1 and 2). GGC retained the characteristics of GC in the lateral scales, dorsal fins and other countable traits. The body length to whole length ratio of GGC was slightly higher than that of KOC and GC. The head width to head length and tail width to tail length ratios of GGC were between those of KOC and GC. In addition, under the SEM, the erythrocytes of GGC, KOC, GC and RCC were analyzed (Fig. 3). The number of erythrocytes in GGC and GC was greater than that in KOC and RCC, while the sizes of the erythrocytes in GGC and GC were smaller than that in KOC and RCC. The erythrocytes nuclear volume (Table 3) ratios of KOC to GGC, GC to GGC and KOC to RCC were 1.91, 0.99 and 1, respectively. The fertilization rate and the hatching rate in GGC were 25 and 18% (Table 4), respectively.

**Table 1:** Comparison of measurable traits among GGC, KOC and GC

Fish type	BL/WL	HL/BL	HW/HL	CPW/CPL	HW/BW	BW/BL
GGC	0.86 ± 0.02	0.22 ± 0.02	0.77 ± 0.04	0.81 ± 0.07	0.75 ± 0.01	0.23 ± 0.01
KOC	0.84 ± 0.04	0.24 ± 0.01	0.82 ± 0.06	0.89 ± 0.04	0.66 ± 0.06	0.30 ± 0.03
GC	0.84 ± 0.01	0.22 ± 0.01	0.70 ± 0.05	0.79 ± 0.05	0.73 ± 0.03	0.21 ± 0.01

**Table 2:** Comparison of countable traits among GGC, KOC and GC

Fish type	No. of lateral scales	No. of upper lateral scales	No. of lower lateral scales	No. of abdominal fins	No. of anal fins	No. of dorsal fins
GGC	41.13 ± 1.36 (39~42)	6	4	III + 7.38 ± 0.48 (7~8)	III + 7	III + 7
KOC	35.67 ± 1.89 (33~37)	6	6	III + 8	III + 6	III + 18.33 ± 0.94 (17~19)
GC	41.5 ± 0.8 (40~43)	6	4	III + 7.79 ± 0.41 (7~8)	III + 7	III + 7

Uppercase Roman numerals indicate the number of the spines, and Arabic numerals reflect the number of soft fins. Numbers before and after the symbol “±” represent the mean value and standard deviation of the fin numbers, respectively. “~” indicates the measured fin number range

**Table 3:** Comparison of nuclear volume among GGC, KOC, GC and RCC in erythrocytes

Fish type	Major semi-axis (μm)	Minor semi-axis (μm)	Volume (μm^3^)
GGC	4.73 ± 0.48	2.98 ± 0.22	42.08 ± 6.75
KOC	5.55 ± 0.25	3.80 ± 0.32	80.51 ± 13.73
GC	4.33 ± 0.31	3.09 ± 0.29	41.64 ± 8.20
RCC	5.37 ± 0.12	3.87 ± 0.08	80.43 ± 1.25

**Table 4:** The fertilization rate and the hatching rate in GGC

Temperature of treatment	No. of eggs	No. of embryos at the gastrula stage	No. of hatching fry	Fertilization rate	Hatching rate
4 °C	73,000	18,251	13,138	25%	18%

### A DNA fragment from the paternal parent is identified in GGC

**Fig. 4:**
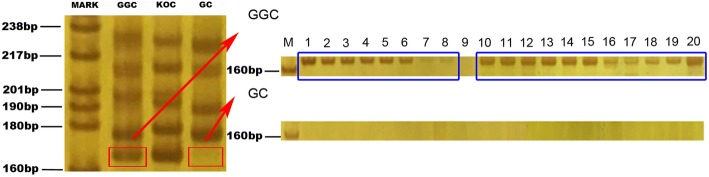
Amplification result of MFW1 in GGC, KOC and GC. MARK (M): The pBR322 DNA/Mspl Marker; GGC: DNA bands amplified from GGC; KOC: DNA bands amplified from KOC; GC: DNA bands amplified from GC; lanes 1~20: Amplification result of MFW1-G in 20 GGC and 20 GC, respectively (the bands amplified in GGC are in blue boxes and no band has been amplified in GC)

**Fig. 5:**
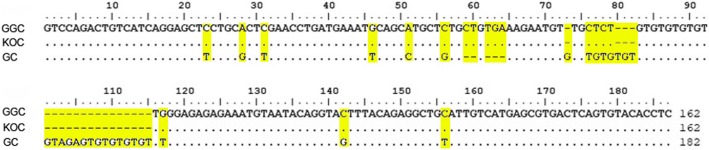
Nucleotide sequence alignment in GGC, KOC and GC (produced by the same primer MFW1). The nucleotide sequence comparisons in the yellow high light indicate that the nucleotide bases in GGC are the same as those of KOC, but are different from those in GC. The dots indicate sequence identity and the hyphens represent insertions/deletions

A pair of microsatellite DNA primers designated MFW1 were used to amplify the DNA fragments from GGC and their parents in this study. Five distinct and bright bands were amplified in GGC by the primers of MFW1. In contrast, only four bands were presented in GC (Fig. 4). After extraction and sequencing, the sequence of MFW1-G, amplified in GGC, was found to be the same (100%) as the sequence amplified in the paternal parent KOC and different from that in GC (Fig. 5). The sequence identity of MFW1-G to the corresponding sequence in common carp (*Cyprinus carpio*, GenBank Accession No. LN590690.1) is 97.5%. The sequence identity of MFW1-G to that of MFW1 in GC (GenBank Accession No. AY703051.1) is 77.4%. The results provided the evidence that MFW1-G in GGC was derived from the paternal parent KOC.

Twenty specimens from GGC and GC respectively were used to repeat the experiments of PCR. According to the results (Fig. 4), MFW1-G was successfully amplified in most of GGC (95%). In contrast, it was not observed in GC, showing the results of the experiments were high consistent.

## Discussion

Artificial gynogenesis is a useful technology for producing the improved species. With this technology, the living gynogenetic progenies have the potential to form improved characteristics in anti-disease ability and immunity ability with the following possible reasons. First, during the process of gynogenesis, the eggs are activated by UV-treated sperm, suggesting that only high-quality eggs can further develop. Thus, the survival gynogenetic progenies have the potential to form stronger characteristics. Second, during the process of gynogenesis, the eggs activated by UV-treated sperm undergo the cold or heat shock, which heavily damage eggs’ development ability, suggesting that the survival gynogenetic progenies have experienced the heavy-stress process. Third, the increased frequencies of some deleterious homologous recessive genes, which makes gynogenetic progenies died. So, the survival gynogenetic progenies have the potential to form improved characteristics. Fourthly, if some paternal genetic materials which insert into the genome or remain in the form of microchromosomes in the gynogenetic progenies, they can result in the “hybrid” effect. In the present study, the gynogenesis hatching rate is very low (18%, Table 4), suggesting that the survival progenies of GGC have the potential to get stronger characteristics. On the other hand, the paternal DNA fragment found in GGC suggests the “hybrid” effect, which possibly makes them stronger in some phenotypes.

It is the first time that MFW1-G has been observed in GGC. The sequence of MFW1-G is the same as that of KOC, and also shows very high homology with that of common carp, suggesting that it might be inherited from KOC. One possibility is that some KOC chromosome fragments including the fragment of MFW1-G are integrated or inserted into the genome of GGC. The other possibility is that the chromosome fragments of KOC have developed into microchromosomes separately, which are not observed in the present study. In this study, the MFW1-G has been amplified by PCR using the primers of MFW1 in GGC with a very high frequency (95%), suggesting that MFW1-G of GGC has been derived from KOC stably. It is possible that the gynogenesis with the DNA fragments derived from the paternal parent in the gynogenetic offspring is a good way to overcome genomic incompatibilities caused by genetic materials from the parents.

## Conclusions

In this study, MFW1-G, a microsatellite DNA fragment, has been observed in GGC, showing the paternal DNA in GGC, which possibly makes GGC stronger in some phenotypes, providing further evidence that the gynogenesis with the DNA fragments derived from the paternal parent in the gynogenetic offspring will be a possible way to get the improved species. The findings of this study are of great significance for fish genetic breeding.

## Abbreviations

BL: Body length
BW: Body width
CPL: Caudal peduncle length
CPW: Caudal peduncle width
GC: Grass carp
GGC: Gynogenetic grass carp
HL: Head length
HW: Head width
KOC: Koi carp
MFW1-G: MFW1-gynogenetic grass carp
PAGE: Polyacrylamide gel electrophoresis
PCR: Polymerase chain reaction
RCC: Red crucian carp
SEM: Scanning electron microscope
SSR: Simple sequence repeats
UV: Ultraviolet
WL: Whole length
