# Correction to: Evidence for paternal DNA transmission to gynogenetic grass carp

**DOI:** 10.1186/s12863-020-00861-3

**Published:** 2020-05-26

**Authors:** Zhuangwen Mao, Yeqing Fu, Yude Wang, Shi Wang, Minghe Zhang, Xin Gao, Kaikun Luo, Qinbo Qin, Chun Zhang, Min Tao, Zhanzhou Yao, Shaojun Liu

**Affiliations:** 1 State Key Laboratory of Developmental Biology of Freshwater Fish, Hunan Normal University, Changsha, 410081 Hunan China; 2 College of Life Sciences, Hunan Normal University, Changsha, 410081 Hunan China

**Correction to: BMC Genet (2019) 20:3**

**https://doi.org/10.1186/s12863-018-0712-x**

Following publication of the original article in *BMC Genetics* [1], the authors detected this error in the discussion on page six: “Third, the increased frequencies of some deleterious homologous recessive genes, which makes gynogenetic progenies died.”

This statement should read: “Third, the increased expressions of some deleterious recessive genes make partial gynogenetic progenies die.”

The authors apologize for any inconvenience caused.
